# *PIEZO1* Hypomorphic Variants in Congenital Lymphatic Dysplasia Cause Shape and Hydration Alterations of Red Blood Cells

**DOI:** 10.3389/fphys.2019.00258

**Published:** 2019-03-15

**Authors:** Immacolata Andolfo, Gianluca De Rosa, Edoardo Errichiello, Francesco Manna, Barbara Eleni Rosato, Antonella Gambale, Annalisa Vetro, Valeria Calcaterra, Gloria Pelizzo, Lucia De Franceschi, Orsetta Zuffardi, Roberta Russo, Achille Iolascon

**Affiliations:** ^1^Department of Molecular Medicine and Medical Biotechnologies, University of Naples Federico II, Naples, Italy; ^2^CEINGE, Biotecnologie Avanzate, Naples, Italy; ^3^Department of Molecular Medicine, University of Pavia, Pavia, Italy; ^4^Pediatric Neurology, Neurogenetics and Neurobiology Unit and Laboratories, Department of Neuroscience, A. Meyer Children’s Hospital, University of Florence, Florence, Italy; ^5^Pediatric Unit, Department of Maternal and Children’s Health, Fondazione IRCCS Policlinico San Matteo, University of Pavia, Pavia, Italy; ^6^Department of Pediatric Surgery, Children’s Hospital “G. Di Cristina”, ARNAS Civico-Di Cristina-Benfretelli, Palermo, Italy; ^7^Department of Medicine, University of Verona, Verona, Italy

**Keywords:** *PIEZO1*, lymphedema, red blood cell alterations, overhydration, stomatocytosis, spherocytosis

## Abstract

PIEZO1 is a cation channel activated by mechanical force. It plays an important physiological role in several biological processes such as cardiovascular, renal, endothelial and hematopoietic systems. Two different diseases are associated with alteration in the DNA sequence of *PIEZO1*: (i) dehydrated hereditary stomatocytosis (DHS1, #194380), an autosomal dominant hemolytic anemia caused by gain-of-function mutations; (ii) lymphatic dysplasia with non-immune fetal hydrops (LMPH3, #616843), an autosomal recessive condition caused by biallelic loss-of-function mutations. We analyzed a 14-year-old boy affected by severe lymphatic dysplasia already present prenatally, with peripheral edema, hydrocele, and chylothoraces. By whole exome sequencing, we identified compound heterozygosity for *PIEZO1*, with one splicing and one deletion mutation, the latter causing the formation of a premature stop codon that leads to mRNA decay. The functional analysis of the erythrocytes of the patient highlighted altered hydration with the intracellular loss of the potassium content and structural abnormalities with anisopoikolocytosis and presence of both spherocytes and stomatocytes. This novel erythrocyte trait, sharing features with both hereditary spherocytosis and overhydrated hereditary stomatocytosis, complements the clinical features associated with loss-of-function mutations of *PIEZO1* in the context of the generalized lymphatic dysplasia of LMPH3 type.

## Background

*PIEZO1* gene encodes for the mechanoreceptor PIEZO1, a selective cation channel activated by mechanical force (Coste et al., 2010), with several different functions, such as regulation of urinary osmolarity (Martins et al., 2016), control of blood pressure (Wang et al., 2016), or sensor of epithelial cell crowding and stretching (Gudipaty et al., 2017). PIEZO1 is expressed in developing blood and lymphatic vessels and plays a key role in blood vessel formation (Andolfo et al., 2013; Li et al., 2014; Ranade et al., 2014). Two different diseases are associated with PIEZO1 mutations: (i) dehydrated hereditary stomatocytosis 1 (DHS1), hemolytic anemia caused by gain-of-function mutations (Zarychanski et al., 2012; Andolfo et al., 2013); (ii) autosomal recessive generalized lymphatic dysplasia with non-immune fetal hydrops (LMPH3) caused by biallelic, loss-of-function mutations (Fotiou et al., 2015; Lukacs et al., 2015). The two diseases are completely different: DHS1 affects red blood cells (RBCs) while LMPH3 is characterized by widespread lymphedema. The only shared phenotype is the presence of perinatal edema (Andolfo et al., 2016; Martin-Almedina et al., 2018).

Several animal models for PIEZO1 were generated. Piezo1-deficient mice die in utero at mid-gestation due to defective vasculogenesis (Cahalan et al., 2015). Thus, another model was developed by a specific deletion in the hematopoietic system (Vav1-P1cKO mice). Interestingly, hematological analysis of Vav1-P1cKO mice revealed elevated MCV and MCH and reduced MCHC (Cahalan et al., 2015). RBCs exhibited increased osmotic fragility, suggesting that Piezo1-deficient erythrocytes were overhydrated. Recently, zebrafish models have also been created. Morpholino-knockdown of Piezo1 expression in *Danio rerio* was reported to result in severe anemia (Faucherre et al., 2014; Shmukler et al., 2015). However, the phenotype observed in the morpholino-knockdown model was not present in an independent zebrafish model carrying a predicted truncated form of Piezo1 (Shmukler et al., 2015). The debate on the phenotype observed in the two different models is still open (Shmukler et al., 2016).

Patients with homozygous loss-of-function mutations in human PIEZO1 show lymphatic dysplasia and an asymptomatic, fully compensated, very mild hemolytic state (Fotiou et al., 2015; Lukacs et al., 2015). Of note, a comprehensible hematological characterization of the anemia carried by patients with *PIEZO1* loss-of-function mutations has not yet been performed. We herein characterized the hematological phenotype of a patient with *PIEZO1* biallelic mutations and lymphatic dysplasia, identifying a new nosological erythrocyte alteration.

## Case Presentation

**FIGURE 1:**
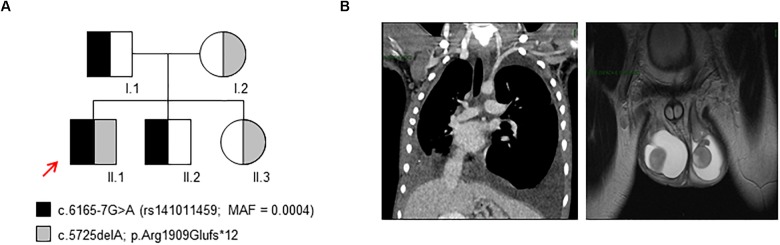
Genetic study and clinical findings. **(A)** The inheritance pattern of c.6165-7G>A and c.5725delA variants in *PIEZO1* in the family here analyzed. The proband (II.1) is a compound heterozygous for these variants. The red arrow indicates the proband. **(B)** Magnetic resonance image of the proband (II.1) showing chylothoraces and hydrocele.

Patient II.1 (Figure 1A) is a 17-years-old male child affected by non-immune hydrops fetalis and congenital lymphatic dysplasia. During pregnancy, a fetal pleural effusion (32 weeks) was observed. The proband was born at 38 weeks by cesarean section. Birth parameters showed a low Apgar score (5/8) with breathing difficulties treated by continuous positive airway pressure, axial hypotonia, peripheral edema, hydrocele, hypoglycemia, and normal auxologic parameters (weight 3.650 Kg; length 53 cm; and head circumference 36 cm). The hemogram resulted normal for age, and total hyperbilirubinemia was observed (13.2 mg/dL) treated by phototherapy. During childhood, a hydrocelectomy (2-years-old) and a scrotum reduction surgery (14-years-old) were performed. At 14 years, a lower limb lymphoscintigraphy was executed, showing distinctive changes of a severe bilateral lymphovascular disease. Particularly, the patient highlighted poor asymmetrical uptake of tracer in the groin at 45 min (almost in the right limb) with evidence of rerouting in the scrotum at 2 h. At 15 years, a thoracentesis was performed to reduce the excess of fluid because of respiratory failure due to restrictive lung disease. The cytological analyses highlighted the presence of chylous fluid. After 1 week the chylous edema was re-observed at X-ray. Due to the worsening of respiratory disease at 16 years, magnetic resonance imaging was performed. The analysis showed an impairment of the chylothoraces and reoccurrence of the hydrocele (Figure 1B). Currently, the proband presents a progressive worsening of the respiratory function.

The other family members are healthy expect for the mother of the proband (I.2) that showed an iron deficiency anemia due to imbalanced diet supplies negative for hemoglobinopathies.

### PIEZO1 Mutational Analysis

We performed WES on the proband and the parents, highlighting the presence of two variants within *PIEZO1* gene: the nucleotide substitution c.6165-7G>A in the intron 42–43, annotated in 1000 Genomes database (rs141011459) with a minor allele frequency (MAF) = 0.0004; the novel nucleotide deletion c.5725delA that results in the frameshift variant p.Arg1909Glufs^∗^12 (Figure 1A). According to the recessive pattern of inheritance, the proband showed a compound heterozygous genotype. Indeed, the father, I.1, carried the variant c.6165-7G>A, while the mother, I.2, carried the variant c.5725delA. We also extended the analysis to additional unaffected subjects: the patient’s brother, II.2, carried the variant c.6165-7G>A, while the sister, II.3, carried the variant c.5725delA.

**FIGURE 2:**
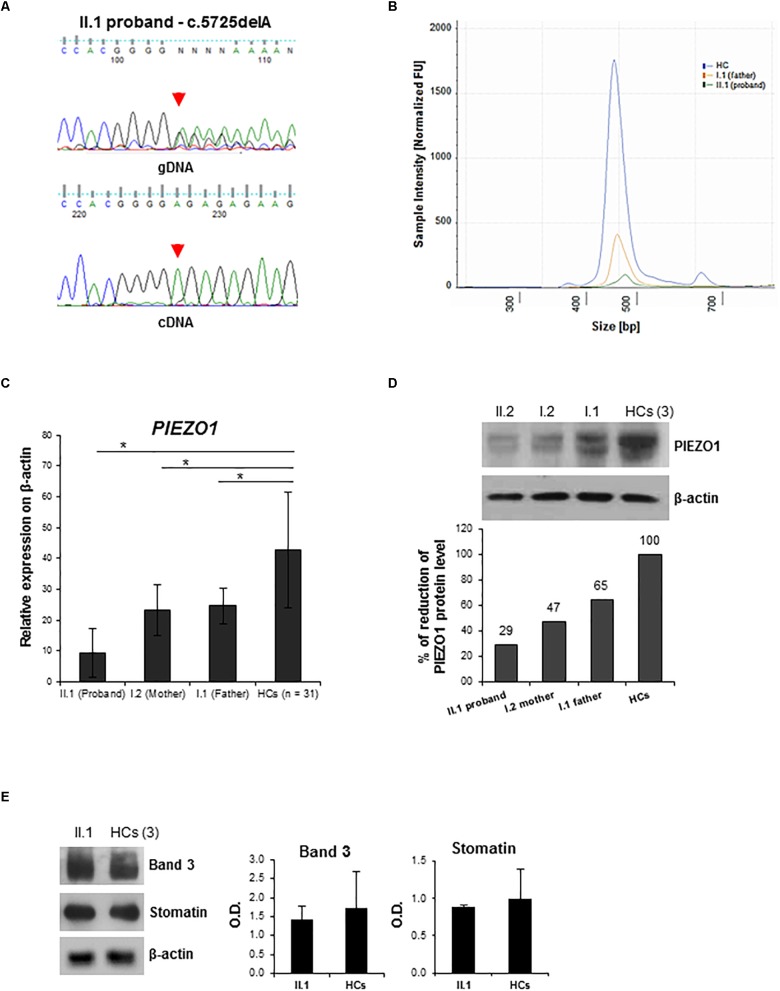
Characterization of *PIEZO1* mutations: cDNA study and membrane proteins expression analysis. **(A)** Electropherograms showing sequencing analysis of the PIEZO1 variant c.5725delA in the proband. Genomic DNA (gDNA) and cDNA sequences are shown. **(B)** DNA electrophoresis profile of the PIEZO1 cDNA fragment encompassing exons 42–44 of the proband (green line), the father (orange line), and the control (blu line) by 4200 TapeStation system. The electropherogram shows the size distribution and the intensity of the detected bands of RT-PCR. **(C)** *PIEZO1* mRNA level normalized to *GAPDH* in the proband II.1 compared to his parents, I.1 and I.2 and the HCs (*n* = 30). Data are presented as a mean ± SD. ^∗^*p*-value < 0.05. **(D)** Immunoblot showing PIEZO1 protein expression normalized to β-actin in the proband II.1 compared to his parents, I.1 and I.2 and the HCs (pool of *n* = 3). Densitometric analysis of one representative western blotting is shown. **(E)** Immunoblot analysis of RBCs membrane proteins, Band 3 (Anion Exchanger 1) and Stomatin (Erythrocyte Membrane Protein 7.2), in the proband II.1 compared to the HCs (pool of *n* = 3). Protein levels are normalized to β-actin. Densitometric analysis of two separate western blotting is shown. Data are presented as a mean ± SD.

To evaluate the possible effect of the frameshift variant on mRNA processing, we sequenced the *PIEZO1* cDNA of the proband. Amplification of the specific exon region, encompassing the mutation, of *PIEZO1* cDNA highlighted the selective expression of the wild-type allele, while the c.5725delA allele was not expressed, demonstrating its decay (Figure 2A). Human Splicing Finder web-tool predicted for the splicing variant c.6165-7G>A the creation of a new “branch point motif,” and two exon splicing enhancer (ESE) motifs for SRp40 protein. High sensitivity analysis of the exon regions encompassing the intronic variant (exons 42–44), using the Agilent 4200 TapeStation system (Supplementary Data Sheet S1), demonstrated that the proband and the father expressed about the 4 and 36%, respectively, of PIEZO1 cDNA compared to the control (Figure 2B).

### Characterization of PIEZO1 Expression

To further evaluate the role of PIEZO1 variants, we assessed gene expression in all the family members, as well as in a subset of healthy controls (HCs). A significant decrease of *PIEZO1* expression in the proband compared to those revealed in the HCs was observed, and a minor decrease (about 50%) of mRNA levels in both parents was detected compared to HCs (Figure 2C). Nevertheless, immunoblot analysis on RBCs membranes highlighted a marked decrease of PIEZO1 protein in the proband compared to the HCs expression with about 30% of expression (Figure 2D). The parents showed also a decrease of PIEZO1 level with 47 and 65% of PIEZO1 expression for mother and father, respectively. Additionally, we evaluated the expression of other RBC membrane proteins, including Band 3 and Stomatin, altered in hereditary spherocytosis (HS) and overhydrated hereditary stomatocytosis (OHS). Proband showed a similar amount of both proteins compared to the HCs (Figure 2E).

### Osmotic Fragility Analysis

**FIGURE 3:**
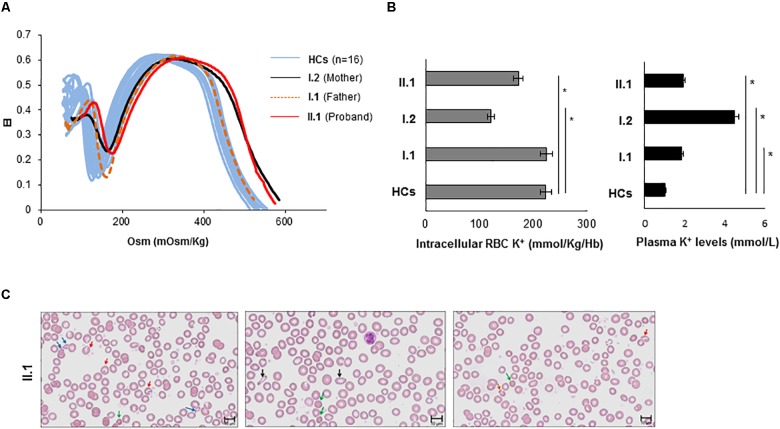
Characterization of the hematological phenotype. **(A)** The red cell deformability index was measured as a function of increasing osmolarity of RBCs from proband II.1 (red line), from mother I.2 (black line), from father I.1 (orange dotted line), and internal HCs (light blue lines). Values are means +/– SE of two independent experiments. Elongation index (EI) **(B)** intracellular K+ content (expressed as mmol/Kg/Hb) of blood from II.1, I.1, I.2 subjects, and from HC (the graph with gray bars). Plasma K+ content (expressed as mmol/L of whole blood) of blood from II.1, I.1, I.2 subjects, and from HC (the graph with black bars) ^∗^*p*-value < 0.05. **(C)** Peripheral blood smear (May-Grünwald Giemsa stain 40×) examination of the proband II.1 showing marked anisopoikolocytosis. Blue arrows indicate fragmented cells; red arrows indicate stomatocytes; green arrows indicate spherocytes; black arrows indicate ovalocytes; orange arrows indicate mushroom cells.

The ektacytometry analysis was performed for the proband and his parents. As shown in Figure 3A, the proband (II.1) exhibited an ektacytometry curve with right shift compared to the curve obtained from the HCs, indicating overhydration of the erythrocytes. The mother (I.2) showed a right shift of the osmolarity curve similar to those observed in the proband. Conversely, the osmolarity curve of the father I.1 was in the range of the controls with a slight right shift of the curve compared to both the proband II.1 and the subject I.2.

### Potassium Content Evaluation

We measured extracellular and intracellular potassium levels in fresh blood samples from all family members, and HCs. The proband (II:1) and his mother (I.2) showed a decrease of potassium content compared to the HC, while the father (I.1) showed intracellular [K+] comparable to HC (Figure 3B). The analysis of K+ plasmatic levels showed increased levels in the proband and his parents compared to the HC.

### Peripheral Blood Smear Examination

The hemogram showed a slight reduction of the Hb content with normal MCV and decreased MCH and MCHC values (Supplementary Table S1). The RDW resulted increased while the reticulocytes count was normal (Supplementary Table S1). Accurate analysis of the peripheral blood (PB) smear of the proband revealed marked anisopoikolocytosis, hypocromia, several spherocytes, some stomatocytes, some mushroom-shaped RBCs, several RBCs fragmentation and debris (Figure 3C).

## Discussion

*PIEZO1* gene encodes for the mechanoreceptor PIEZO1, a selective cation channel activated by mechanical force (Coste et al., 2010; Kim et al., 2012; Ge et al., 2015; Gnanasambandam et al., 2015; Andolfo et al., 2016; Dubin et al., 2017; Hyman et al., 2017; Zhao et al., 2017). In human, the first disease associated with mutations in *PIEZO1* was the DHS1 (Zarychanski et al., 2012; Andolfo et al., 2013). In erythrocytes, PIEZO1 regulates cell volume homeostasis, and gain-of-function mutations in DHS1 are causative of alterations of the RBC membrane permeability to monovalent cations Na+ and K+, with consequent alterations of the intracellular cationic content and cell volume (Albuisson et al., 2013; Bae et al., 2013; Archer et al., 2014; Sandberg et al., 2014; Shmukler et al., 2014; Imashuku et al., 2016). Generally, DHS1 patients show hemolytic anemia, with high reticulocyte count, the tendency to macrocytosis, and mild jaundice (Zarychanski et al., 2012; Andolfo et al., 2018b). The second condition associated with *PIEZO1* mutations is the lymphatic dysplasia. Two recent reports have described homozygous or compound heterozygous mutations in *PIEZO1* in families with LMPH3 (Fotiou et al., 2015; Lukacs et al., 2015). These cases exhibited full body edema and severe facial swelling. Most patients also presented intestinal lymphangiectasia, growth retardation, seizures, microcephaly, and intellectual disability. Loss-of-function mutations in *PIEZO1* also account for hydrops fetalis, chylothorax, and chronic pleural effusions with persistent lymphedema of legs, torso, and face. The cosegregating homozygous and compound heterozygous *PIEZO1* mutations in these families included non-sense, missense, and splice donor site mutations (Fotiou et al., 2015; Lukacs et al., 2015). Regarding the hematological framework, some of these patients were not anemic and exhibited normal hematological indices, including MCV (Lukacs et al., 2015).

The patient herein described shared some similar characteristics with the other LMPH3 patients until described such as hydrops fetalis, chylothorax, and chronic pleural effusions with persistent lymphedema. On the other hand, our patient showed peculiar characteristics: the hydrocele never observed in the other *PIEZO1* loss-of-function patients, and the absence of facial swelling, lymphangiectasia, and intellectual disability. Of note, the proband is a compound heterozygous for a splicing variant and a coding deletion that causes a premature stop codon. We demonstrated the decay of the allele carrying the deletion variant, and the massive reduction of expression of the allele carrying the splicing variant. The combination of the two variants causes a substantial reduction of both mRNA and protein expression of PIEZO1 in the proband.

*PIEZO1* is a highly polymorphic gene that has a very large tolerance for both missense and loss-of-function variants and has a lot of variations. The variable expressivity of both DHS1 and lymphatic dysplasia could be explained with the combination of multiple disease-causing alleles or their combination with polymorphic variants (Lupski, 2012; Lacroix et al., 2018). Indeed, we previously demonstrated that multiple modifier *PIEZO1* variants could account for highly variable clinical expressivity in DHS1, with subsequent difficulties in establishing the appropriate genotype/phenotype correlation (Andolfo et al., 2018a,b). Of note, the patient showed a peculiar phenotype characterized by peripheral edema, hydrocele, and chylothoraces. Furthermore, even if the blood count seems only slightly altered with a mild reduction of the Hb, and decreased MCH and MCHC values, the RDW resulted increased despite the reticulocytes count was normal. According to the increased RDW, the PB smear of the proband revealed anisopoikolocytosis, hypocromia, with the presence of some spherocytes, mushroom-shaped RBCs, stomatocytes, erythrocytes’ fragmentation, and debris. Moreover, the ektacytometry analysis revealed a right shift of the right arm of the osmolarity curve indicating mild overhydration of RBCs, without the decreased DImax typical of HS. Finally, the ionic flux assay indicated increased plasma [K+] and decreased intracellular [K+] as in OHS. Thus, our patient seems to present pathological traits of the erythrocyte with some characteristics shared with hereditary spherocytosis as spherocytes at PB smear and normal MCV and several features of overhydrated hereditary stomatocytosis as stomatocytes at PB, decreased MCHC, normal Dimax, right shift of the osmolarity curve, and decreased intracellular potassium. The mother showed a similar, but less pronounced, right shift of the osmolarity curve. This finding could be caused by the iron deficiency anemia that is known to alter the deformability of RBCs (Vayá et al., 2005; Brandão et al., 2009).

Of note, Vav1-P1cKO mice with specific deletion of *Piezo1* in the hematopoietic system showed a slight increase of RDW and reduced MCHC confirming overhydration of RBCs as seen in our patient (Cahalan et al., 2015). Moreover, morpholino-knockdown of Piezo1 in zebrafish showed the erythroid phenotype of fragile, spherocytic, dysmorphic cells also like our patient (Shmukler et al., 2015).

In conclusion, the proband presents an alteration of the structure and the ionic content of erythrocytes caused by the two hypomorphic variants in *PIEZO1*. We speculate that the substantial decreased expression of PIEZO1 could be compensated by overactivation of other cation channels/pumps that act by compensating the hematological phenotype. Patients affected by lymphedema caused by mutations in *PIEZO1* could benefit in future of therapy by Yoda1, a novel small synthetic molecule specific activator of PIEZO1 (Cahalan et al., 2015; Lacroix et al., 2018), or by gene therapy by selective insertion of the gene in the lymphatic system, or by *in vivo* target gene activation via CRISPR/CAS9 mediated *trans*-epigenetic modulation.

## Data Availability

All datasets generated for this study are included in the manuscript and/or the Supplementary Files.

## Ethics Statement

Ethics Committee of University Federico II, number 197/18.

## Author Contributions

IA, RR, and AI designed and conducted the study, and prepared the manuscript. GDR performed the western blotting analysis and contributed to the preparation of the manuscript. EE and AV performed the preparation of the WES libraries and the NGS analysis. FM and BER performed the molecular analysis and collection of the samples. AG, VC, and GP contributed to take care of the patients. LDF performed the ionic flux data analysis. RR performed the mutational analysis. OZ designed and supervised the NGS analysis and also provided a critical evaluation of the study.

## Conflict of Interest Statement

The authors declare that the research was conducted in the absence of any commercial or financial relationships that could be construed as a potential conflict of interest.

## References

[B1] Albuisson J. Murthy S. E. Bandell M. Coste B. Louis-Dit-Picard H. Mathur J. (2013). Dehydrated hereditary stomatocytosis linked to gain-of-function mutations in mechanically activated piezo1 ion channels. *Nat. Commun.* 4:1884. 10.1038/ncomms2899 23695678 PMC3674779

[B2] Andolfo I. Alper S. L. De Franceschi L. Auriemma C. Russo R. De Falco L. (2013). Multiple clinical forms of dehydrated hereditary stomatocytosis arise from mutations in PIEZO1. *Blood* 121 3925–3935. 10.1182/blood-2013-02-482489 23479567

[B3] Andolfo I. Manna F. De Rosa G. Rosato B. E. Gambale A. Tomaiuolo G. (2018a). PIEZO1-R1864H rare variant accounts for a genetic phenotype-modifier role in dehydrated hereditary stomatocytosis. *Haematologica* 103 e94–e97. 10.3324/haematol.2017.180687 29191841 PMC5830381

[B4] Andolfo I. Russo R. Rosato B. E. Manna F. Gambale A. Brugnara C. (2018b). Genotype-phenotype correlation and risk stratification in a cohort of 123 hereditary stomatocytosis patients. *Am. J. Hematol.* 93 1509–1517. 10.1002/ajh.25276 30187933

[B5] Andolfo I. Russo R. Gambale A. Iolascon A. (2016). New insights on hereditary erythrocyte membrane defects. *Haematologica* 101 1284–1294. 10.3324/haematol.2016.142463 27756835 PMC5394881

[B6] Archer N. M. Shmukler B. E. Andolfo I. Vandorpe D. H. Gnanasambandam R. Higgins J. M. (2014). Hereditary xerocytosis revisited. *Am. J. Hematol.* 89 1142–1146. 10.1002/ajh.23799 25044010 PMC4237618

[B7] Bae C. Gnanasambandam R. Nicolai C. Sachs F. Gottlieb P. A. (2013). Xerocytosis is caused by mutations that alter the kinetics of the mechanosensitive channel PIEZO1. *Proc. Natl. Acad. Sci. U.S.A.* 110 E1162–E1168. 10.1073/pnas.1219777110 23487776 PMC3606986

[B8] Brandão M. M. Castro Mde L. Fontes A. Cesar C. L. Costa F. F. Saad S. T. (2009). Impaired red cell deformability in iron deficient subjects. *Clin. Hemorheol. Microcirc.* 43 217–221. 10.3233/CH-2009-1211 19847056

[B9] Cahalan S. M. Lukacs V. Ranade S. S. Chien S. Bandell M. Patapoutian A. (2015). Piezo1 links mechanical forces to red blood cell volume. *eLife* 22:4. 10.7554/eLife.07370 26001274 PMC4456639

[B10] Coste B. Mathur J. Schmidt M. Earley T. J. Ranade S. Petrus M. J. (2010). Piezo1 and Piezo2 are essential components of distinct mechanically activated cation channels. *Science* 330 55–60. 10.1126/science.1193270 20813920 PMC3062430

[B11] Dubin A. E. Murthy S. Lewis A. H. Brosse L. Cahalan S. M. Grandl J. (2017). Endogenous piezo1 can confound mechanically activated channel identification and characterization. *Neuron* 94 266–270. 10.1016/j.neuron.2017.03.039 28426961 PMC5448662

[B12] Faucherre A. Kissa K. Nargeot J. Mangoni M. E. Jopling C. (2014). Piezo1 plays a role in erythrocyte volume homeostasis. *Haematologica* 99 70–75. 10.3324/haematol.2013.086090 23872304 PMC4007942

[B13] Fotiou E. Martin-Almedina S. Simpson M. A. Lin S. Gordon K. Brice G. (2015). Novel mutations in PIEZO1 cause an autosomal recessive generalized lymphatic dysplasia with non-immune hydrops fetalis. *Nat. Commun.* 6:8085. 10.1038/ncomms9085 26333996 PMC4568316

[B14] Ge J. Li W. Zhao Q. Li N. Chen M. Zhi P. (2015). Architecture of the mammalian mechanosensitive Piezo1 channel. *Nature* 527 64–69. 10.1038/nature15247 26390154

[B15] Gnanasambandam R. Bae C. Gottlieb P. A. Sachs F. (2015). Ionic Selectivity and Permeation Properties of Human PIEZO1 Channels. *PLoS One* 8:e0125503. 10.1371/journal.pone.0125503 25955826 PMC4425559

[B16] Gudipaty S. A. Lindblom J. Loftus P. D. Redd M. J. Edes K. Davey C. F. (2017). Mechanical stretch triggers rapid epithelial cell division through Piezo1. *Nature* 543 118–121. 10.1038/nature21407 28199303 PMC5334365

[B17] Hyman A. J. Tumova S. Beech D. J. (2017). Piezo1 channels in vascular development and the sensing of shear stress. *Curr. Top Membr.* 2017 37–57. 10.1016/bs.ctm.2016.11.001 28728823

[B18] Imashuku S. Muramatsu H. Sugihara T. Okuno Y. Wang X. Yoshida K. (2016). PIEZO1 gene mutation in a Japanese family with hereditary high phosphatidylcholine hemolytic anemia and hemochromatosis-induced diabetes mellitus. *Int. J. Hematol.* 104 125–129. 10.1007/s12185-016-1970-x 26971963

[B19] Kim S. E. Coste B. Chadha A. Cook B. Patapoutian A. (2012). The role of *Drosophila* piezo in mechanical nociception. *Nature* 483 209–212. 10.1038/nature10801 22343891 PMC3297676

[B20] Lacroix J. J. Botello-Smith W. M. Luo Y. (2018). Probing the gating mechanism of the mechanosensitive channel Piezo1 with the small molecule yoda1. *Nat. Commun.* 9:2029. 10.1038/s41467-018-04405-3 29795280 PMC5966384

[B21] Li J. Hou B. Tumova S. Muraki K. Bruns A. Ludlow M. J. (2014). Piezo1 integration of vascular architecture with physiological force. *Nature* 515 279–283. 10.1038/nature13701 25119035 PMC4230887

[B22] Lukacs V. Mathur J. Mao R. Bayrak-Toydemir P. Procter M. Cahalan S. M. (2015). Impaired PIEZO1 function in patients with a novel autosomal recessive congenital lymphatic dysplasia. *Nat. Commun.* 6:8329. 10.1038/ncomms9329 26387913 PMC4578306

[B23] Lupski J. R. (2012). Digenic inheritance and mendelian disease. *Nat. Genet.* 44 1291–1292. 10.1038/ng.2479 23192179

[B24] Martin-Almedina S. Mansour S. Ostergaard P. (2018). Human phenotypes caused by piezo1 mutations one gene, two overlapping phenotypes? *J. Physiol.* 596 985–992. 10.1113/JP275718 29331020 PMC5851881

[B25] Martins J. R. Penton D. Peyronnet R. Arhatte M. Moro C. Picard N. (2016). Piezo1-dependent regulation of urinary osmolarity. *Pflugers Arch.* 468 1197–1206. 10.1007/s00424-016-1811-z 27023350

[B26] Ranade S. S. Qiu Z. Woo S.-H. Hur S. S. Murthy S. E. Cahalan S. M. (2014). Piezo1, a mechanically activated ion channel, is required for vascular development in mice. *Proc. Natl. Acad. Sci. U.S.A.* 111 10347–10352. 10.1073/pnas.1409233111 24958852 PMC4104881

[B27] Sandberg M. B. Nybo M. Birgens H. Frederiksen H. (2014). Hereditary xerocytosis and familial haemolysis due to mutation in the piezo1 gene: a simple diagnostic approach. *Int. Jnl. Lab Hematol.* 36 e62–e65. 10.1111/ijlh.12172 24314002

[B28] Shmukler B. E. Huston N. C. Thon J. N. Ni C. W. Kourkoulis G. Lawson N. D. (2015). Homozygous knockout of the piezo1 gene in the zebrafish is not associated with anemia. *Haematologica* 100 e483–e485. 10.3324/haematol.2015.132449 26294733 PMC4666336

[B29] Shmukler B. E. Lawson N. D. Paw B. H. Alper S. L. (2016). Authors response to “comment on: homozygous knockout of the piezo1 gene in the zebrafish is not associated with anemia”. *Haematologica* 101:e39. 10.3324/haematol.2015.137810 26721803 PMC4697908

[B30] Shmukler B. E. Vandorpe D. H. Rivera A. Auerbach M. Brugnara C. Alper S. L. (2014). Dehydrated stomatocytic anemia due to the heterozygous mutation R2456H in the mechanosensitive cation channel PIEZO1: a case report. *Blood Cells Mol. Dis.* 52 53–54. 10.1016/j.bcmd.2013.07.015 23973043

[B31] Vayá A. Simó M. Santaolaria M. Todolí J. Aznar J. (2005). Red blood cell deformability in iron deficiency anaemia. *Clin. Hemorheol. Microcirc.* 33 75–80. 16037635

[B32] Wang S. Chennupati R. Kaur H. Iring A. Wettschureck N. Offermanns S. (2016). Endothelial cation channel PIEZO1 controls blood pressure by mediating flow-induced ATP release. *J. Clin. Invest.* 126 4527–4536. 10.1172/JCI87343 27797339 PMC5127677

[B33] Zarychanski R. Schulz V. P. Houston B. L. Maksimova Y. Houston D. S. Smith B. (2012). Mutations in the mechanotransduction protein piezo1 are associated with hereditary xerocytosis. *Blood* 120 1908–1915. 10.1182/blood-2012-04-422253 22529292 PMC3448561

[B34] Zhao Q. Wu K. Chi S. Geng J. Xiao B. (2017). Heterologous expression of the Piezo1-ASIC1 chimera induces mechanosensitive currents with properties distinct from Piezo1. *Neuron* 94 274–277. 10.1016/j.neuron.2017.03.040 28426963

