# Supplementary material for: PIEZO1 Hypomorphic Variants in Congenital Lymphatic Dysplasia Cause Shape and Hydration Alterations of Red Blood Cells
Source: Front Physiol. 2019 Mar 15;10:258. doi: 10.3389/fphys.2019.00258 (PMC6428731; doi:10.3389/fphys.2019.00258)
Supplement: Supplementary file 1 [file Data_Sheet_1.pdf]

Supplemental data

PIEZO1 hypomorphic variants in congenital lymphatic dysplasia cause shape and hydration alterations of red blood cells

Immacolata Andolfo[1,2], Gianluca De Rosa[1,2], Edoardo Errichiello[3], Francesco Manna[1,2], Barbara Eleni Rosato[1,2], Antonella Gambale[1,2], Annalisa Vetro[4], Valeria Calcaterra[5], Gloria Pellizzo[6], Lucia De Franceschi[7], Orsetta Zuffardi[3], Roberta Russo[1,2], Achille Iolascon[1,2].

Corresponding author:
Immacolata Andolfo, PhD
Department of Molecular Medicine and Medical Biotechnologies
University of Naples, Federico II, 80145, Naples, Italy
CEINGE, Biotecnologie Avanzate,
Via Gaetano Salvatore, 486, 80145, Naples, Italy
Tel: +39-081-3737736
Fax: +39-081-3737804
e-mail: andolfo@ceinge.unina.it

Material and Methods

Collection of patients and genetic testing

Five subjects from one Italian family were enrolled in the study. The clinical data of the index case are summarized in Table S1, as obtained by the referral clinicians of the patient and with the approval of local university ethical committees. The clinical diagnosis of lymphatic dysplasia was based on history, clinical findings, and radiological data. DNA was obtained for genetic analysis from affected and unaffected family members, and healthy controls (HCs) after signed informed consent, according to the Declaration of Helsinki. Written informed consent was obtained from the participants for the publication of this case report. The protocol was approved by the ethical committee of University Federico II of Naples.

Reads from whole-exome sequencing (WES) were aligned to the most recent version of the human genome (GRCh37/hg19) using the BWA software package v0.5.9 (17) (see supplemental data for details).

Whole-exome capture and sequencing

Reads were aligned to the most recent version of the human genome (GRCh37/hg19) using the BWA software package v0.5.9 as previously described[17]. WES analysis was performed by analyzing the DNA of the patient and the parents, on Hiseq2500 sequencer (Illumina). Sequencing was preceded by selective enrichment of the regions coding of the human genome (SureSelectXT Clinical Research Exome kit, Agilent Technologies). The average coverage of the region's target was greater than 80 readings per nucleotide in the proband and 55 readings per nucleotide in the parents. Direct sequencing analysis of the additional family members was performed as previously described[17]. The filtering of the gene variants was performed using a dedicated analysis pipeline that considered non-synonymous exonic variants or sites splicing, and not reported in dbSNP138 or in ESP with a frequency in the general population of more than 1%. The variants thus obtained were manually reviewed to verify the quality of the data and analyzed in the light of the HGMD database and the Mutation Taster prediction tools, SIFT and Polyphen2.

RNA isolation, cDNA preparation, and quantitative RT-PCR

Total RNA was extracted from patients and healthy controls (HC n=31) peripheral blood using Trizol reagent (Life Technologies). cDNA synthesis from total RNA (2 µg) was performed using SuperScript II First Strand kits (Life Technologies). Quantitative RT-PCR (qRT-PCR) was performed by the SYBR-green method, following standard protocols with an Applied Biosystems

ABI PRISM 7900HT Sequence Detection system. Relative gene expression was calculated using the $2^{(-\Delta Ct)}$ method, where ΔCt indicates the differences in the mean Ct between selected genes and the normalization control (β-actin). qRT-PCR primers for each gene were designed as previously described[17].

PIEZO1 exons 42-44 coding sequence was amplified by KAPA2G Robust HotStart ReadyMix (Kapa Biosystems, Cape Town, South Africa). Sequence primers are available on request. RT-PCR was quantified using the Agilent 4200 TapeStation with Agilent D1000 ScreenTape Assay (Agilent Technologies, CA).

Immunoblotting

RBCs membrane extracts (80 µg protein) were loaded on SDS-polyacrylamide gels, transferred onto polyvinylidene difluoride membranes (BioRad, Milan, Italy) and incubated with the following antibodies: rabbit anti-PIEZO1 (1:500; Proteintech), rabbit anti-Band 3 (1:200; Santa-Cruz Biotech), rabbit anti-Stomatin (1:1000; Genetex). Mouse anti-β-actin antibody (1:12000; Sigma Aldrich, Milan, Italy) was used as a control for the equal loading. Incubation with HRP-conjugated anti-rabbit Ig (1:4000) (GE Healthcare, UK) and HRP-conjugated anti-mouse Ig (1:4000) (GE Healthcare, UK) was performed and labeled bands were visualized (Supersignal West Pico Chemiluminescent Substrate Kit, ThermoScientific, Miami USA). Densitometric analysis was performed with the BioRad Chemidoc using Quantity One software (BioRad).

Osmotic gradient ektacytometry

Deformability of the RBCs of the patients and relative control subjects were evaluated by osmotic gradient ektacytometry using the Laser-assisted Optical Rotational Cell Analyzer (LORCA) as previously described[17].

Measurements of potassium ion fluxes in red blood cells from patients

The erythrocytes of the affected and unaffected family members were treated as previously described[17]. The free hemoglobin (Hb) levels were measured to evaluate the degree of hemolysis as for potassium. Cell and medium K content, as well as cell Rb content, were determined in triplicate by atomic absorption spectroscopy (ANALYST 2000, Perkin-Elmer) as previously described[17] and normalized on PIEZO1 protein quantity.

Statistical analysis

Data are presented as a mean ± standard deviation (SD) or standard error (SE). Statistical significance was calculated using the Student's t-test. $P<0.05$ was considered as statistically significant.
