# Supplementary material for: PIEZO1 Hypomorphic Variants in Congenital Lymphatic Dysplasia Cause Shape and Hydration Alterations of Red Blood Cells
Source: Front Physiol. 2019 Mar 15;10:258. doi: 10.3389/fphys.2019.00258 (PMC6428731; doi:10.3389/fphys.2019.00258)
Supplement: Supplementary file 2 [file Table_1.docx]

**Table S1. Clinical and genetic findings of family members**

|  | II.1 | I.1 | I.2 | Normal range* | | |
| --- | --- | --- | --- | --- | --- | --- |
|  |  |  |  | *Child* | *Adult male* | *Adult female* |
| *PIEZO1 genotype*^§^ | c.6165-7G>A/c.5725delA, p.Arg1909Glufs*12 | wt/c.6165-7G>A | wt/c.5725delA, p.Arg1909Glufs*12 | - | - | - |
| *Neonatal history* | NIHF; breathing difficulties; peripheral edema; hydrocele | - | - | - | - | - |
| *Lymphedema* | Bilateral lymphovascular limb disease; chylothoraces; hydrocele; worsening of the respiratory function | - | - | - | - | - |
| *Complete blood count* |  |  |  |  |  |  |
| RBC (10^6^/µl) | 5.06 | 5.13 | 4.47 | 3.9-5.6 | 4.2-5.6 | 4.0-5.4 |
| Hb (g/dl) | 12.5 | 15.3 | 12.6 | 11.0-16.0 | 12.0-17.5 | 12.0-16.0 |
| MCV (fl) | 83.2 | 86.7 | 71.8 | 70.0-91.0 | 80.0-97.0 | 80.0-97.0 |
| MCH (pg) | 25.3 | 29.8 | 29.2 | 23.0-33.0 | 25.0-34.0 | 25.0-34.0 |
| MCHC (g/dl) | 30.1 | 34.4 | 27.6 | 23.0-33.0 | 32.0-38.0 | 32.0-38.0 |
| Ret (%) | 1.6 | - | - | 0.5-2 | 0.5-2 | 0.5-2 |
| RDW (%) | 17.6 | 12.7 | 15.4 | 11-16 | 11-16.5 | 11-16.5 |
| *Laboratory data* |  |  |  |  |  |  |
| Total bilirubin (mg/dl) | 0.5 | *-* | - | 0.2-1.2 | 0.2-1.2 | 0.2-1.2 |
| LDH (U/L) | 195 | - | - | 125.0-243.0 | 125.0-243.0 | 125.0-243.0 |

*Reference ranges from AOU Federico II, University of Naples, Italy.

^§^NM_001142864; NP_001136336.
